# Surface Chloride Concentration of Concrete under Shallow Immersion Conditions

**DOI:** 10.3390/ma7096620

**Published:** 2014-09-15

**Authors:** Jun Liu, Kaifeng Tang, Dong Pan, Zongru Lei, Weilun Wang, Feng Xing

**Affiliations:** Guangdong Provincial Key Laboratory of Durability for Marine Civil Engineering, College of Civil Engineering, Shenzhen University, Shenzhen 518060, Guangdong, China; E-Mails: tangkaifeng@email.szu.edu.cn (K.T.); 2011090158@email.szu.edu.cn (D.P.); 2012090303@email.szu.edu.cn (Z.L.); wang_weilun@hotmail.com (W.W.)

**Keywords:** surface chloride concentration, shallow immersion, concrete, fly ash, pore structure

## Abstract

Deposition of chloride ions in the surface layer of concrete is investigated in this study. In real concrete structure, chloride ions from the service environment can penetrate into concrete and deposit in the surface layer, to form the boundary condition for further diffusion towards the interior. The deposit amount of chloride ions in the surface layer is normally a function of time, rather than a constant. In the experimental investigation, concrete specimens with different mix proportions are immersed in NaCl solution with a mass concentration of 5%, to simulate the shallow immersion condition in sea water, and the surface chloride concentrations are measured at different ages. It is found that the surface chloride concentration increases following the increasing immersion durations, and varies from a weight percentage of 0.161%–0.781% in concretes with different mix proportions. The w/c (water-to-cement ratio) influences the surface chloride concentration significantly, and the higher the w/c is, the higher the surface chloride concentration will be, at the same age. However, following the prolonging of immersion duration, the difference in surface chloride concentration induced by w/c becomes smaller and smaller. The incorporation of fly ash leads to higher surface chloride concentration. The phenomena are explained based on pore structure analyses.

## 1. Introduction

Plenty of steel reinforced concrete structures are constructed under chloride environments, such as marine/coastal environment, deicing salt environment, industrial waste water environment, *etc*. Under such environments, chloride ions can easily penetrate into concrete, as concrete is a porous material due to its porous binder phase and imperfect interfaces [1,2]. Chloride ions have to deposit in the surface layer of concrete first, and then diffuse into the interior. When the chloride concentration at the depth of steel reinforcement reaches a threshold, steel corrosion is initiated [3,4,5,6,7,8]. Propagation of steel corrosion will lead to structural deterioration and certainly shorten the service life of the structure. Chloride induced steel corrosion has been considered as one of the most significant reasons for structural failure all over the world [9]. In steel reinforced concrete structure, concrete cover is designed to protect steel bars from corrosion, thus the effect of the service environment on the structure has to begin from the surface layer of concrete. In chloride environments, chloride ions penetrate into and deposit in the surface layer of concrete to form the boundary condition for further diffusion into the interior concrete, and the denser surface layer may retard the ingress of chloride ions and prolong the service life of the structure [10]. According to Fick’s second law, the ingress of chloride ions is governed by two factors, *i.e.*, the surface chloride concentration and the chloride diffusion coefficient of the matrix. The former is the focus of the present study.

To describe the behavior of chloride in concrete, Fick’s second law of diffusion normally needs to be solved. This law in the case of one-dimensional diffusion can be expressed as:

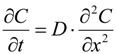
(1)
where *C* is the chloride concentration; *t* is the diffusion time; *D* is the diffusion coefficient; and *x* is the depth from the diffusion boundary. Assuming that *D* is a constant without changing with time, and that chloride concentration at the boundary is initially and always keeps *C_s_*, under the initial condition that *C*(*x*, 0) = 0, the analytical solution of Equation (1) can be derived as:

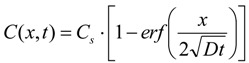
(2)
where *erf*() is the error function. In a marine environment, a concrete structure may include three kinds of components, *i.e.*, submerged structure, splash zone and superstructure. According to Amey *et al*. [11], transport of chloride ions in submerged or splash zone components can be described by Equation (2), as in which *C_s_* can be considered as a constant equal to the chloride concentration of sea water. However, when Equation (1) is used in air borne (superstructure) or deicing salt applications, *C_s_* cannot be simplified as a constant, but a function of time, *i.e.*,

*C_s_* = *C*(0,*t*) = Φ(*t*) (3)

To describe the buildup of the chloride concentration at the surface of concrete, different functions can be assigned to Φ(*t*). If linear relation is employed, *i.e.*, Φ(*t*) = *kt* where *k* is a constant, the solution of Equation (1) can be written as [11]:

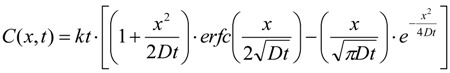
(4)
where *erfc*() is the complementary error function. If the square root relation is chosen, *i.e.*, 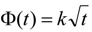, the analytical solution of Equation (1) can be derived as:

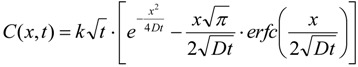
(5)


Other researchers also proposed exponential relation between *C_s_* and *t*, *i.e.*, *C_s_* = *C*_*s*0_ · (1 − *e*^–*at*^), based on experimental observations and statistical analyses [12]. Furthermore, as investigated by McGee [13], the concrete surface chloride concentration is a function of the distance between the target structure and the coastline (*d*). When *d* is smaller than 0.1 km, *C_s_* equals to 2.85 kg Cl^−^/m^3^ concrete; when *d* is between 0.1 km and 2.84 km, *C_s_* = 1.15–1.81 lg(*d*) kg Cl^−^/m^3^ concrete; when *d* is greater than 2.84 km, *C_s_* = 0.03 kg Cl^−^/m^3^ concrete. It means that structures located within 100 m distance from the coastline have the highest risk, due to the highest surface chloride concentration. Considering the typical density of concrete, *i.e.*, 2400 kg/m^3^, the 2.85 kg Cl^−^/m^3^ concrete can be transformed to 0.12% of the mass of concrete, which seems to be inappropriately low. According to an investigation involving 2384 concrete samples taken from 487 positions of 94 piers in Hong Kong [14], in almost all cases, the chloride concentration at the depth of steel bars can reach the threshold value that is set for inducing the corrosion of steel bars, *i.e.*, 0.06% of the mass of concrete. As estimated from the results through analytical models, the surface chloride concentration should be from 0.25% to 0.30%, which is consistent with the Bamforth’s suggestion [15] and the value for superstructure components in marine structures as suggested by a Chinese technical report “Guide for durability based design and construction of concrete structure” [16]. 

Although the values introduced above, as either reported or suggested, are named surface chloride concentration, they are not the real values of the surface concentrations. The surface chloride concentrations are normally determined by averaging on a thin surface layer, e.g., the chloride concentration in the layer within 1 inch below the surface is averaged, considered as the concentration at the depth of 13 mm and used as the surface chloride concentration [11,17]. Actually, even in the submerged and splash zone components, the surface chloride concentration does not equal the concentration of sea water due to the binding of chloride ions in concrete, and is difficult to determine due to the difficulty in defining the real “surface”. A surface layer as thin as possible should be used to determine the surface chloride concentration resulted from the deposition of chloride ions. Under such a strategy, in the present study, well-cured concrete specimens are immersed in NaCl solution, to simulate the service environment of immersion in shallow sea water. The evolution of the surface chloride concentration, as influenced by w/c (water-to-cement ratio), age and the incorporation of fly ash, is investigated. The discussion is limited to the shallow immersion condition, in which the diffusion can be considered as the sole mechanism of chloride ingress. The microstructures are also investigated in order to understand the said influences, in light of mercury intrusion porosimetry (MIP) and scanning electron microscopy (SEM).

## 2. Experimental Section

### 2.1. Materials

A CEM I Portland cement supplied by Shenzhen Haixing Onoda Cement Co. Ltd., Shenzhen, China, was used as the cementitious material in the preparation of concrete mixture. A Class F fly ash supplied by Mawan Power Plant, Shenzhen, China, was used as the supplementary cementitious material, to partially replace cement in some concrete mixtures. The chemical compositions of the cement and fly ash, expressed as mass percentages of oxides, are listed in Table 1. Other materials involved in concrete preparation included distilled water, river sand with the fineness modulus of 2.61 and the apparent density of 2632 kg/m^3^ as fine aggregate, and gravel with the particle size of 5–20 mm and the apparent density of 2700 kg/m^3^ as coarse aggregate. Sodium chloride with a purity above 99% was used to prepare NaCl solution that was used for immersing the concrete specimens.

**Table 1:** Chemical composition of cement (mass%).

Materials	CaO	SiO_2_	Al_2_O_3_	Fe_2_O_3_	MgO	SO_3_	K_2_O	LOI
Cement	64.67	18.59	4.62	4.17	2.35	3.32	0.92	1.03
Fly ash	4.74	62.32	23.95	1.33	2.04	1.25	0.76	3.12

### 2.2. Experiments

Fresh concrete mixtures were mixed according to the mix proportions listed in Table 2, where the mix proportions are expressed in masses of ingredients in 1 m^3^ of fresh concrete. Sand takes a volume fraction of 40% in aggregate of mixes PC53, PC47 and PC38, in which the binder consists of plain Portland cement and the w/c equals 0.53, 0.47 and 0.38, respectively. Mixes FC47-15 and FC47-30 are obtained through replacing the cement in PC47 by fly ash by mass fractions of 15% and 30% respectively, thus the w/b (water-to-binder mass ratio) of both the two fly ash incorporated concrete mixtures are 0.47 too. After they are mixed, the mixtures were cast in steel molds with the dimensions of 100 × 100 × 100 mm^3^, and covered with plastic sheets. After 24 h, all specimens were de-molded and cured in a moisture room where the temperature and relative humidity are 20 ± 2 °C and >95%, respectively.

**Table 2:** Mix proportions of concrete specimens (kg/m^3^ fresh concrete).

Mix	w/b	Water	Cement	Fly Ash	Sand	Gravel
PC53	0.53	201	379	0	720	1079
PC47	0.47	192	409	0	720	1079
PC38	0.38	175	461	0	720	1079
FC47-15	0.47	191	346	61	715	1072
FC47-30	0.47	190	283	121	711	1065

**Figure 1:**
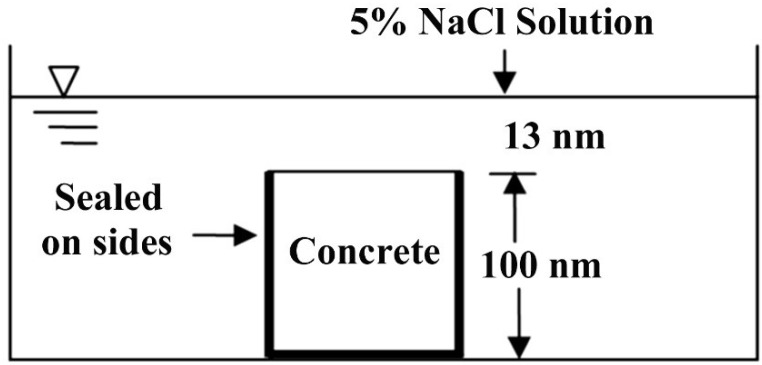
Sketch of the immersion experiment.

After a 30-day curing, the concrete specimens were taken out from the curing room, sealed by paraffin wax on five plane faces, and immersed in 5% (mass concentration) NaCl solution with the unsealed face upwards, as shown in Figure 1. The concentration of 5% is higher than the mean concentration of sea water, but it is normally used in researches, for the purpose of advisable acceleration [17]. The solution surface was 13 mm higher than the upper surfaces of the specimens. The volume ratio of liquid to solid specimens was maintained at approximately 3 for the immersions, and no refreshment of the liquid were made during the immersion period. After the immersion durations of 30, 60, 90, 120 and 150 days, the chloride ion content accumulated in the surface layer was determined by chemical titration, according to a Chinese standard “Testing Code of Concrete for Port and Waterwog Engineering (JTJ 270-98)” [18]. To do so, the surface layer with a thickness of 3 mm was cut and ground into powder, and water-soluble extraction was performed afterward. The chloride ions concentration in the leachate was measured by AgNO_3_ titration using a Metrohm 809 Titrando automatic potentiometric titrator, and then transformed to the chloride content of the surface layer concrete in mass percentage. Average values calculated over three parallel tests were reported as results in this study. It is worth noting that the chloride content detected in this way is the water-soluble chloride content, chemically bonded chloride ions exclusive, as the concentration gradient of water soluble chloride (or free chloride) is the real driving force for chloride diffusion.

Mercury intrusion porosimetry (MIP), which has been widely used in materials science [19,20,21], was employed to characterize the changing of pore structure of concrete following increasing immersion ages. For MIP tests, surface layer (within 3 mm from the surface) of concrete specimens, both before and after immersion, were sawed off and crushed. After the elimination of large aggregate particles, small pieces were taken as samples. The samples for MIP test were dried following the solvent (ethanol) replacement drying procedure [19]. Based on the general suggestions in the literature [21], in the theoretical calculation of pore diameter by the well-known Washburn equation, the contact angle between mercury and hardened cement paste was chosen as 130°, while the surface tension of mercury was 480 mN/m. A Micromeritics AutoPore IV 9500 was used for MIP tests, and the maximum pressure that could be applied is 30,500 psi (210 MPa), which approximately corresponds to a minimum detectable pore diameter of 6 nm. 

Scanning electron microscopy (SEM) was employed to observe the microscopic morphology of the concrete specimens. For this, aged concrete specimens were crushed into small pieces. After the elimination of aggregate particles, the pieces of concrete were dried under 50 °C to constant weights, and coated with a thin gold layer right before being put into the microscope.

## 3. Results

### 3.1. Influence of w/c on the Surface Chloride Concentration

**Figure 2:**
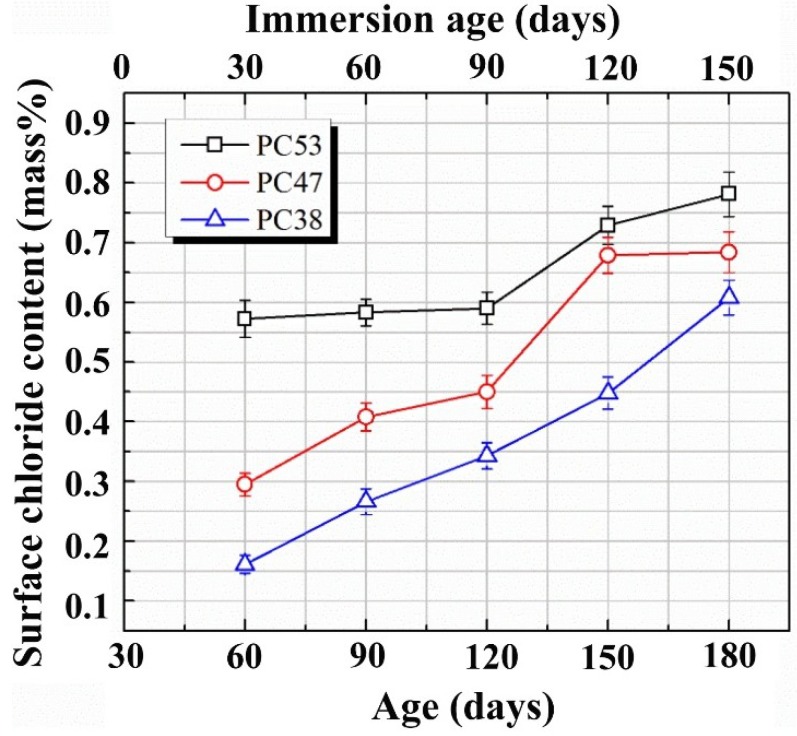
Evolutions of the surface chloride concentrations of plain cement concretes as immersed in NaCl solution.

Evolutions of the surface chloride concentrations in the plain cement concretes, PC53, PC47 and PC38, as determined in the proposed experiments, are plotted in Figure 2 *versus* the immersion duration. From Figure 2, it is clear that in plain cement concretes, the changing ranges of the surface chloride concentration are 0.572%–0.781%, 0.295%–0.684% and 0.161%–0.608% when the w/c equals 0.53, 0.47 and 0.38, respectively. For each w/c, the surface chloride concentration increases following the increasing immersion durations, and at each time point, the higher the w/c is, the higher the surface chloride concentration will be. For example, after a 30-day immersion, the surface chloride concentration of PC53 is 0.572d, which is 1.94 times that of PC47 and 3.55 times that of PC38. However, the difference between concretes with different w/c becomes smaller gradually following the increase of the immersion duration, so that after the immersion for 150 days, the value of PC53 is only 1.14 times that of PC47 and 1.28 times that of PC38.

**Figure 3:**
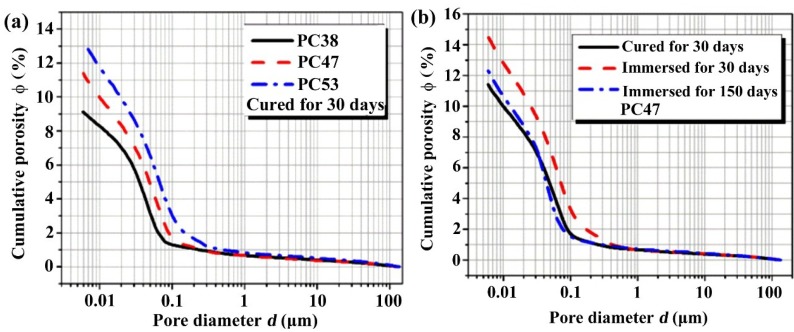
Pore structures of plain cement concretes characterized by MIP: (**a**) influence of w/c, after cured for 30 days; (**b**) influence of immersion.

There are different mechanisms governing the evolution of surface chloride concentration in plain cement concrete, including penetration and deposition of chloride ions, cement hydration and leaching of cement hydrates (mainly calcium hydroxide). Chloride penetration and deposition make the chloride amount higher and higher in the surface layer. Cement hydration results in more and more hydrates and lower and lower porosity. On the one hand, more hydrates bind more chloride ions; on the other hand, lower porosity means less pore solution carrying chloride ions, thus cement hydration may lower down the surface chloride concentration, as measured by the method used in this study. Leaching, which makes higher surface porosity and higher pore network connectivity [17,22], can undoubtedly bring on higher surface chloride concentration. It is the competition of these mechanisms which results in the overall trends as shown in Figure 2. As only water-soluble chloride content was measured, the surface chloride content may be governed by the surface porosity, as higher surface porosity means more pore solution carrying free chloride. The higher the w/c, the higher the capillary porosity, as proven by the MIP results in Figure 3a, and thus the higher the surface chloride concentration. The above-mentioned effects of leaching on the pore structure is proven by Figure 3b, where it can also be found that the negative effect of leaching cannot be redeemed due to the continuous hydration even until the immersion age of 150 days. It has been proven that when the w/c is high, continuous hydration can keep in force until very late age, but when the w/c is low, the degree of hydration may reach its ultimate level at a relatively early age [17]. That is why the late age increasing rate of the surface chloride concentration in the low w/c concrete seems to be higher than that in high w/c concrete as illustrated by the plots in Figure 2. 

### 3.2. Influence of Fly Ash on the Surface Chloride Concentration

**Figure 4:**
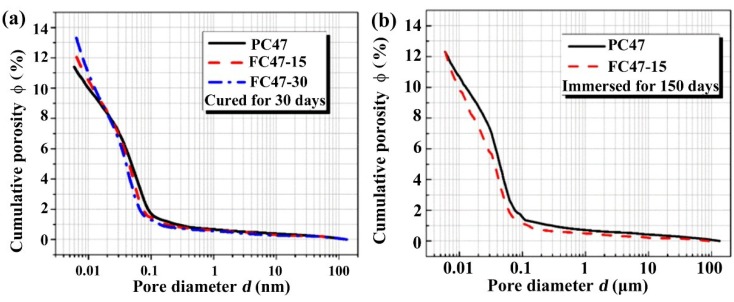
Pore structures of fly ash incorporated concretes characterized by MIP: (**a**) influence of fly ash, after cured for 30 days; (**b**) comparison after long term immersion.

The replacement of cement by fly ash results in lower initial porosity of cement paste with the same w/b, as the specific gravity of fly ash is much lower than Portland cement [23]. However, the reaction of fly ash does not start until sometime after mixing. In the case of Class F fly ash, this can be as long as one week or even longer. From the overall view of the binder system, the hydration/reaction process is retarded [23,24,25,26]. As a result, the incorporation of fly ash leads to higher capillary porosity and coarser pore structure at an early age. This inverse influence cannot be redeemed even after a curing of 30 days, as shown in Figure 4a. However, once the pozzolanic reaction of the fly ash is initialized due to the high alkalinity supplied by cement hydration, it will progress continuously to form secondary calcium silicate hydrates (C–S–H) and other crystlline reaction products, and the degree of reaction depends on the degree of hydration of cement [23]. Under the effects of the pozzolanic reaction, unless the glass content of fly ash is too low, fly ash will help to achieve comparable capillary porosity and a much more tortuous pore network, as compared with plain cement concrete with the same w/b, as far as the long-term pore structure is considered. This trend is valid even when leaching is considered under the shallow immersion condition, as shown in Figure 4b. 

**Figure 5:**
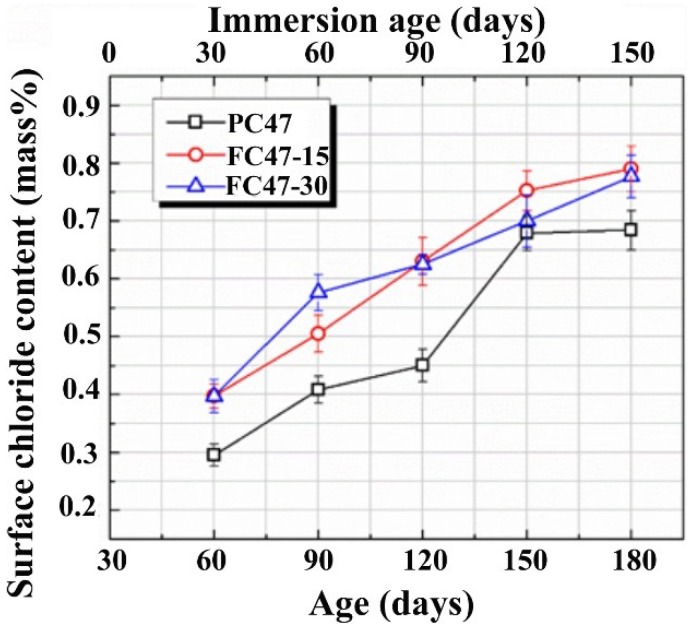
Evolutions of the surface chloride contents of pure cement concrete and fly ash incorporated concretes as they are immersed in NaCl solution.

As shown in Figure 4, the incorporation of fly ash brings changes to the microstructure of the surface layer of concrete. These micro-structural changes certainly result in changes in the surface chloride contents. The evolutions of surface chloride contents in plain cement concrete PC47 and the fly ash incorporated concretes with the same w/b are compared in Figure 5. It is a common feature that the surface chloride content increases following the increasing immersion durations, due to the above-mentioned mechanisms governing the evolution of surface chloride content. As the capillary porosities of the surface layers of fly ash incorporated concretes are higher than that of the plain cement concrete, as shown in Figure 4a, the surface chloride contents are also higher, due to the existence of more pore solution accommodating free chloride ions. However, comparing the curves of FC47-15 and FC47-30, it can be concluded that higher replacement ratio of fly ash does not bring further higher surface chloride concentration than the lower replacement ratio case. From Figure 5, it can also be seen that the difference between surface chloride contents of the plain cement concrete and the fly ash incorporated concretes tends to become smaller, as they are immersed in the NaCl solution. This may be attributed to the long-term pozzolanic reaction of fly ash, which results in more significant late age microstructural changes, as proven by Figure 4.

### 3.3. SEM

**Figure 6:**
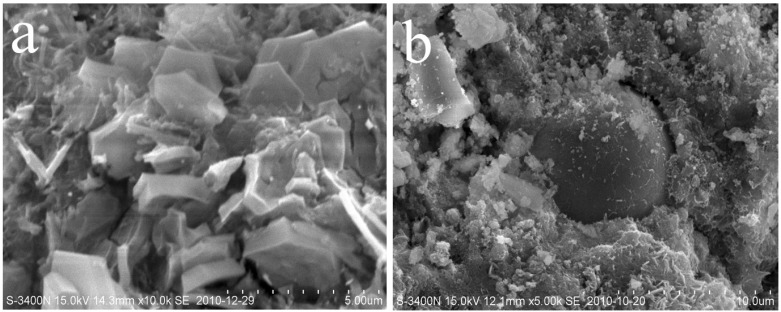
Microscopic morphology of concrete at the immersion age of 150 days as observed by SEM: (**a**) PC47; (**b**) FC47-15.

The macroscopic behavior of a material is determined by its microstructure [27,28]. The MIP results shown above have explained some of the behaviors of the concretes. SEM provides a direct observation of the microscopic morphology of concrete. The microstructures of PC47 and FC47-15 at the immersion age of 150 days, as obtained by SEM, are shown in Figure 6. In the PC47, as shown in Figure 6a, large crystals can be observed. These hexagonal-slice-shaped crystals are Friedel’s salt formed due to the reaction between chloride and aluminate cement hydrates [17]. Figure 6b illustrates the characteristics of the surface layer of fly ash incorporated concrete, except for the formation of the Friedel’s salt. As shown in Figure 6b, the degree of reaction of fly ash is low, leading to the higher capillary porosity; impenetrable fly ash particles are wrapped by penetrable C-S-H gel, resulting in the more tortuous pore structure.

### 3.4. Discussion

The surface chloride concentration, as determined by the method used in the present study, is an average concentration of a surface layer, or the concentration at a fictitious surface. Thus, it cannot be directly used in Fick’s second law based models to predict the chloride profile in a target concrete. Through observations on Figure 2 and Figure 5, it seems that the buildup of the surface chloride concentration in the shallow immersed concrete can be described approximately by a linear Φ(*t*). Thus, under the studied environment, chloride profile after a specific immersion duration can be predicted by using Equation (4). A necessary modification is that the depth index *x* should be replaced by *x−x*_0_, where *x*_0_ denotes the depth of the fictitious surface and equals 1.5 mm in the present study.

## 4. Conclusions

In the present study, the deposition of chloride ions in the surface layer of concrete under shallow immersion, as expressed by surface chloride content, is investigated. The effects of w/c and fly ash incorporation are evaluated, and the mechanisms behind these effects are revealed in light of pore structure measurements and microscopic observation. Through this study, several conclusions can be drawn as follows:

(1) Under real service conditions, the surface chloride concentration of concrete structures is not a constant, but a function of time.
(2) In a concrete under shallow immersion, the surface chloride concentration increases with the increase of the immersion duration.
(3) In plain cement concretes, the higher the w/c, the higher the surface chloride concentration. However, the difference becomes smaller with the increasing immersion durations.
(4) The incorporation of fly ash leads to higher surface chloride concentration as compared with the reference plain cement concrete with the same w/b.

## References

[1] Ma H., Hou D., Lu Y., Li Z. (2014). Two-scale modeling of the capillary network in hydrated cement paste. Constr. Build. Mater..

[2] Ma H., Li Z. (2014). Multi-aggregate approach for modeling interfacial transition zone in concrete. ACI Mater. J..

[3] Al Mutlaq F.M., Page C.L. (2013). Effects of electric arc furnace dust on susceptibility of steel to corrosion in chloride-contaminated concrete. Constr. Build. Mater..

[4] Poupard O., Aït-Mokhtar A., Dumargue P. (2004). Corrosion by chlorides in reinforced concrete: Determination of chloride concentration threshold by impedance spectroscopy. Cem. Concr. Res..

[5] Shafei B., Alipour A., Shinozuka M. (2012). Prediction of corrosion initiation in reinforced concrete members subjected to environmental stressors: A finite-element framework. Cem. Concr. Res..

[6] Yuan Q., Shi C., de Schutter G., Audenaert K., Deng D. (2009). Chloride binding of cement-based materials subjected to external chloride environment: A review. Constr. Build. Mater..

[7] Angst U., Elsener B., Larsen C.K., Vennesland Ø. (2009). Critical chloride content in reinforced concrete: A review. Cem. Concr. Res..

[8] Glass G.K., Buenfeld N.R. (1997). The presentation of the chloride for corrosion of steel in threshold concrete. Corros. Sci..

[9] Vaysburd A.M., Emmons P.H. (2000). How to make today’s repairs durable for tomorrow—Corrosion protection in concrete repair. Constr. Build. Mater..

[10] Horgnies M., Willieme P., Gabet O. (2011). Influence of the surface properties of concrete on the adhesion of coating: Characterization of the interface by peel test and FT-IR spectroscopy. Prog. Org. Coat..

[11] Amey S.L., Johnson D.A., Miltenberger M.A., Farzam H. (1998). Predicting the service life of concrete marine structure: An environmental methodology. ACI Struct. J..

[12] Kassir M.K., Ghosn M. (2002). Chloride-induced corrosion of reinforced concrete bridge decks. Cem. Concr. Res..

[13] McGee R. Modelling of durability performance of tasmanian bridges. Proceedings of ICASP8 Applications of Statistics and Probability in Civil Engineering.

[14] Tavwood Engineering Ltd. (1996). Condition Audit of Reinforced Concrete Piers and Review of Concrete Design for the Marine Environment Executive Summary.

[15] Bamforth P.B. (1998). Spreadsheet model for reinforcement corrosion in structures exposed to chloride. Concrete Under Severe Condition.

[16] Chen Z., Zhao G. (2004). Guide for Durability Based Design and Construction of Concrete Structure.

[17] Liu J., Xing F., Dong B., Ma H., Pan D. (2013). Study on water sorptivity of the surface layer of concrete. Mater. Struct..

[18] Ministry of Transport of the People’s Republic of China (1998). JTJ 270-98. Testing Code of Concrete for Port and Waterwog Engineering.

[19] Ma H., Li Z. (2013). Realistic pore structure of Portland cement paste: Experimental study and numerical simulation. Comput. Concr..

[20] Ma H., Xu B., Lu Y., Li Z. (2014). Modelling magnesia-phosphate cement paste at the micro-scale. Mater. Lett..

[21] Ma H. (2014). Mercury intrusion porosimetry in concrete technology: Tips of measurement, pore structure parameter acquisition and application. J. Porous Mater..

[22] Liu J., Xing F., Dong B., Ma H., Pan D. (2014). Study on surface permeability of concrete under immersion. Materials.

[23] Ma H. (2013). Multi-Scale Modeling of the Microstructure and Transport Properties of Contemporary Concrete. Ph.D. Thesis.

[24] Narmluk M., Nawa T. (2011). Effect of fly ash on the kinetics of Portland cement hydration at different curing temperatures. Cem. Concr. Res..

[25] Fraay A.L., Bijen J.M., de Haan Y.M. (1989). The reaction of fly ash in concrete: A critical examination. Cem. Concr. Res..

[26] Liu J., Qiu Q., Xing F., Pan D. (2014). Permeation properties and pore structure of surface layer of fly ash concrete. Materials.

[27] Hou D., Ma H., Zhu Y., Li Z. (2014). Calcium silicate hydrate from dry to saturated state: Structure, dynamics and mechanical properties. Acta Mater..

[28] Neville A.M. (1996). Properties of Concrete.

